# Purification and Characterization of the RecA Protein from *Neisseria gonorrhoeae*

**DOI:** 10.1371/journal.pone.0017101

**Published:** 2011-02-17

**Authors:** Elizabeth A. Stohl, Marielle C. Gruenig, Michael M. Cox, H. Steven Seifert

**Affiliations:** 1 Department of Microbiology-Immunology, Northwestern University Feinberg School of Medicine, Chicago, Illinois, United States of America; 2 Department of Biochemistry, University of Wisconsin-Madison, Madison, Wisconsin, United States of America; Institut Pasteur, France

## Abstract

The strict human pathogen *Neisseria gonorrhoeae* is the only causative agent of the sexually transmitted infection gonorrhea. The *recA* gene from *N. gonorrhoeae* is essential for DNA repair, natural DNA transformation, and pilin antigenic variation, all processes that are important for the pathogenesis and persistence of *N. gonorrhoeae* in the human population. To understand the biochemical features of *N. gonorrhoeae* RecA (RecA_Ng_), we overexpressed and purified the RecA_Ng_ and SSB_Ng_ proteins and compared their activities to those of the well-characterized *E. coli* RecA and SSB proteins *in vitro*. We observed that RecA_Ng_ promoted more strand exchange at early time points than RecA_Ec_ through DNA homologous substrates, and exhibited the highest ATPase activity of any RecA protein characterized to date. Further analysis of this robust ATPase activity revealed that RecA_Ng_ is more efficient at displacing SSB from ssDNA and that RecA_Ng_ shows higher ATPase activity during strand exchange than RecA_Ec_. Using substrates created to mimic the cellular processes of DNA transformation and pilin antigenic variation we observed that RecA_Ec_ catalyzed more strand exchange through a 100 bp heterologous insert, but that RecA_Ng_ catalyzed more strand exchange through regions of microheterology. Together, these data suggest that the processes of ATP hydrolysis and DNA strand exchange may be coupled differently in RecA_Ng_ than in RecA_Ec_. This difference may explain the unusually high ATPase activity observed for RecA_Ng_ with the strand exchange activity between RecA_Ng_ and RecA_Ec_ being more similar.

## Introduction

RecA or RecA-like proteins are ubiquitous in nearly all cells and are essential for the processes of homologous recombination, recombinational DNA repair, induction of the SOS response of DNA repair, SOS mutagenesis, and chromosome partitioning [1]. All these processes are crucial for maintenance of genome stability and survival of DNA damage. *In vitro*, *E. coli* RecA is a DNA-dependent ATPase that promotes a three DNA strand-exchange reaction between homologous double-stranded DNA (dsDNA) and circular single-stranded DNA (cssDNA), which is believed to mimic the recombination functions of RecA *in vivo* [2], [3]. DNA strand exchange occurs in three distinct steps. First, in the presence of an adenine nucleotide cofactor, RecA binds ssDNA in a 5′ to 3′ direction, forming an active helical nucleoprotein filament; second, the nucleoprotein filament aligns with homologous dsDNA, forming a joint molecule; third, the region of hybrid DNA is extended via unidirectional branch migration, yielding nicked circular dsDNA and the displaced linear ssDNA product. *E. coli* RecA-mediated ATP hydrolysis occurs throughout a nucleoprotein filament [4], and is required for disassembly from the 5′-proximal end [5]–[7]. The processes of homologous pairing and strand exchange between short regions of homologous DNA (∼1 kb) require ATP binding but not ATP hydrolysis [5], [8]–[10]. However, ATP hydrolysis is required to exchange longer homologous DNA substrates because it is coupled to the unidirectional extension phase of DNA strand exchange [11], and ATP hydrolysis is also required to bypass barriers of heterology [12], [13]. Finally, *E. coli* RecA exhibits a coprotease activity. In the context of an active nucleoprotein filament, RecA facilitates the self-cleavage of the LexA repressor protein, leading to induction of over 30 genes of the *E. coli* SOS regulon [14]. RecA also facilitates cleavage of the UmuD protein and participates directly in mutagenic translesion DNA synthesis [15]–[17].

The strand exchange catalyzed by *E. coli* RecA is strongly stimulated by the *E. coli* SSB protein [18] as well as other SSB orthologs [19]–[21], and *E. coli* SSB functions with other RecA proteins [22], [23]. SSB is a heterotetrameric, single-stranded DNA binding protein that melts secondary structure in ssDNA, thereby allowing RecA monomers to form an active nucleoprotein filament [24]. SSB also functions postsynaptically, binding the displaced single strand of DNA [25]. However, when present in a reaction before RecA, SSB presents a barrier to RecA nucleation, which RecA overcomes only slowly *in vitro* [26]–[29]. *In vivo*, it is likely that RecA relies heavily on the activity of recombination mediator proteins for loading onto SSB-coated DNA [26].

The obligate human pathogen *N. gonorrhoeae* (the gonococcus, Gc) is a Gram-negative diplococcus and is the sole causative agent of the sexually transmitted infection gonorrhea. In *N. gonorrhoeae* homologous DNA recombination mediated by RecA is required for recombinational DNA repair, as well as the cellular processes of natural DNA transformation and pilin antigenic variation, and *recA* is essential for all of these processes [27], [28]. RecA mediates recombinational DNA repair in *N. gonorrhoeae* in concert with either the RecBCD or RecF-like pathway (so designated because it lacks a RecF homologue) [29]–[32]. Natural DNA transformation in *N. gonorrhoeae* refers to the ability to take up gonococcal DNA from the environment in a sequence-dependent manner and incorporate it into its genome [33]–[35]. Many gene products are involved in DNA uptake and transformation competence, including factors involved in the elaboration of type IV pili [36]. Once DNA is inside the cell, it is recombined into the chromosome, a process which requires RecA, PriA, and is partially dependent on the RecBCD, Rep, and RecN enzymes [28], [29], [31], [37], [38]. The ability of *N. gonorrhoeae* to incorporate foreign DNA aids in the transfer of antibiotic resistance genes [39] and contributes to the antigenic diversity of the population [40], both of which are important for the continued spread and persistence of Gc in the human population.

The plasticity of the gonococcal genome is perhaps best exemplified by the process of pilin antigenic variation, which is also dependent on *recA* [27]. Antigenic variation occurs via unidirectional homologous recombination between the pilin coding gene (*pilE*) and one of many unexpressed *pilS* loci located at several sites on the gonococcal chromosome. This recombination can occur with as little as 2–4 bases of sequence identity [41], [42], suggesting that antigenic variation is a specialized form of homologous recombination. Pilin antigenic variation occurs quite frequently, at a rate of 4×10^−3^ events per cell per generation [42]. Although antigenic variation does not require the RecBCD pathway [43], in addition to RecA antigenic variation requires the RecF-like pathway [29], the branch migration enzymes RuvABC and RecG [44], the RdgC [45], and Rep proteins [37]. Antigenic variation of the gonococcal pilus is believed to be an important means of evasion of the host immune response, aiding in successful transmission of the organism. Therefore, the gonococcal RecA is central to the pathogenesis of *N. gonorrhoeae*, catalyzing recombinational DNA repair, contributing to the spread of antibiotic resistance genes, and catalyzing antigenic variation of the pilus.

*N. gonorrhoeae* has historically been reported to lack a classical SOS response [46] due the absence of UmuC, and UmuD homologues and dearth of canonical SOS boxes in the genome [47]. Moreover, neither the *recA* transcript nor RecA protein levels increase after treatment of *N. gonorrhoeae* with DNA damaging agents [47], [48]. Although in *E. coli*, the LexA response regulator represses the expression of approximately 40 SOS genes [49], recent work has revealed that *N. gonorrhoeae* encodes a LexA homologue that controls the expression of a small gene regulon [50].

The importance of the *recA* gene for *N. gonorrhoeae* pathogenesis has been recognized for nearly 20 years [27], [28]. Although there has been extensive analysis of the genetic requirements for these cellular processes, biochemical characterization of the proteins involved in these processes has lagged behind. The parsed amino acid sequence of the *N. gonorrhoeae* RecA shows a high degree of overall sequence identity with the *E. coli* RecA protein (65% identity; 81% similarity); however, the sequences diverge in their carboxy termini [51], a variability that has been noted in other bacterial RecA proteins [52]. Despite the difference in sequence, the abundance of negatively charged residues in the *E. coli* RecA C-terminal 25 amino acids is conserved in *N. gonorrhoeae*. These C-terminal sequence differences may be important for the interaction of the RecA proteins with other cellular proteins, or may be important for the intrinsic activities of the RecA proteins. Therefore, we purified the *N. gonorrhoeae* RecA and SSB proteins (RecA_Ng_ and SSB_Ng_) to assess the recombinase, ATPase, and coprotease activities of RecA_Ng_ compared to those of the well-characterized *E. coli* RecA protein. We found that RecA_Ng_ exhibits the highest ATPase activity of any characterized RecA protein, but catalyzes only slightly more strand exchange at early time points than RecA_Ec_.

## Results

### Experimental rationale

*N. gonorrhoeae* RecA is essential for the diverse cellular processes of DNA repair, pilin antigenic variation, and natural DNA transformation. We therefore characterized the *N. gonorrhoeae* RecA and SSB proteins to determine whether they possess unique biochemical properties that could account for the importance of recombination-based processes in the lifestyle of this obligate human pathogen.

### Identification and cloning of the *N. gonorrhoeae ssb* gene

**Figure 1:**
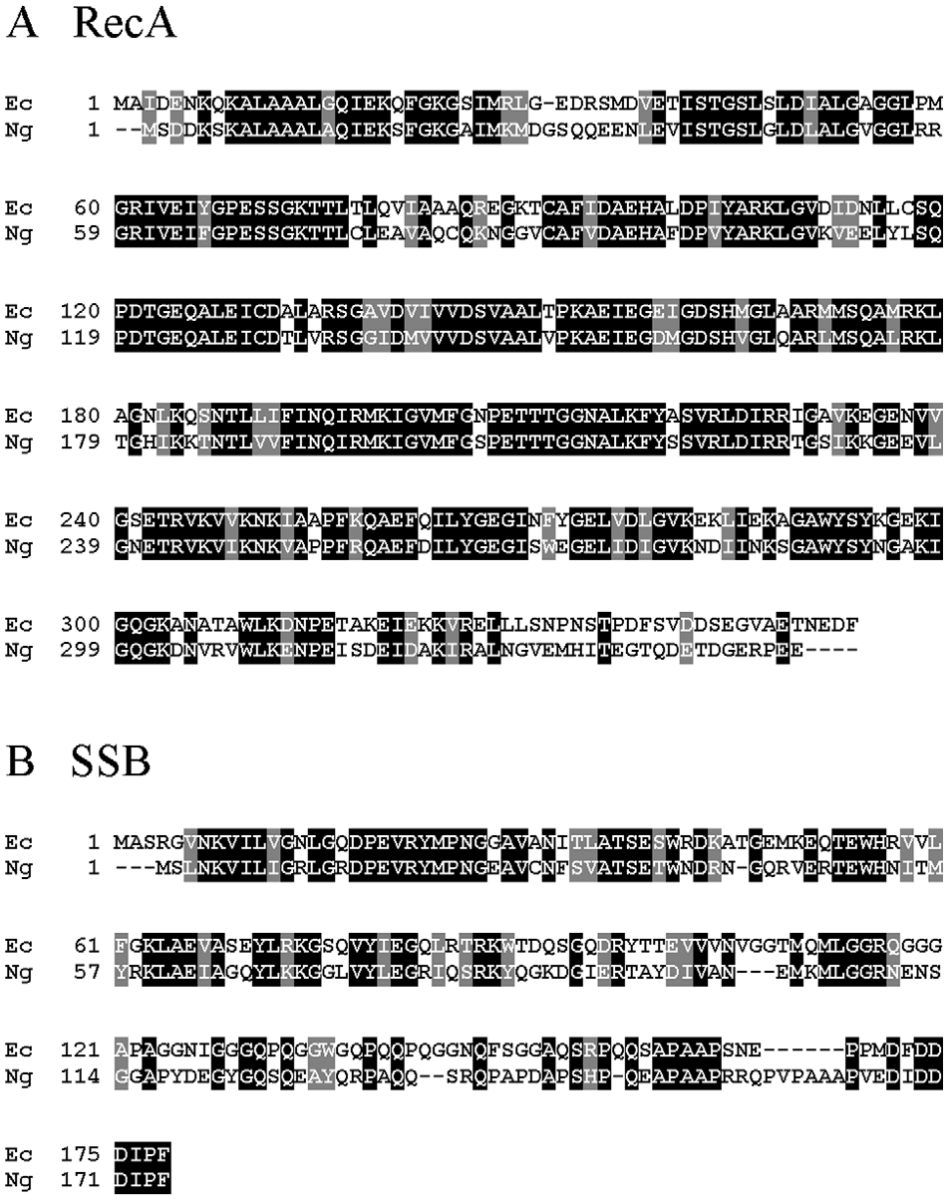
Amino acid sequence alignments of RecA and SSB proteins from *E. coli* (Ec) and *N. gonorrhoeae* (Ng). Identical residues are boxed in black; similar residues are boxed in grey. Dashes represent gaps introduced to optimize sequence alignment. A. Alignment of RecA proteins. B. Alignment of SSB proteins.

To identify open reading frames in the *N. gonorrhoeae* strain FA1090 genome with sequence similarity to the *E. coli ssb* gene, we performed a search of the FA1090 genome using the STDGEN website (http://stdgen.northwestern.edu/). A single open reading frame (ORF) was identified which was predicted to encode a 167 amino acid protein showing 63% sequence similarity and 50% sequence identity to the 169-amino acid *E. coli* SSB (SSB_Ec_) protein (Figure 1) over the length of the protein. This ORF was amplified from FA1090 chromosomal DNA and cloned into expression vector pET21a. The nucleotide sequence of the amplified *ssb* gene was identical to the sequence in the STDGEN website.

### Purification of the recombinant RecA_Ng_ and SSB_Ng_ proteins from *E. coli*

The RecA_Ng_ and SSB_Ng_ proteins (Figure 1) were both overexpressed in *E. coli* and purified to >99.9% homogeneity (Figure S1 and data not shown). Following the basic purification strategy for *E. coli* RecA (see Materials and Methods), we noted subtle differences in the purification profiles of RecA_Ng_ versus RecA_Ec_. First, in the DEAE-Sepharose column purification step, RecA_Ng_ bound to the column, whereas RecA_Ec_ is found in the initial column flow-through. Second, RecA_Ng_ required a higher concentration of phosphate to elute from the hydroxyapatite column than does RecA_Ec_. The purity of RecA_Ng_ was assessed by SDS-polyacrylamide gel electrophoresis and Coomassie blue staining (Figure S1). Purified RecA_Ng_ migrated slightly faster than RecA_Ec_ on SDS-PAGE (data not shown), which is consistent with the predicted difference in molecular masses of the two proteins (37,842 Da for *E. coli* versus 37,700 Da for *N. gonorrhoeae* RecA) (Figure 1).

### RecA_Ng_ shows increased recombinase activity relative RecA_Ec_

**Figure 2:**
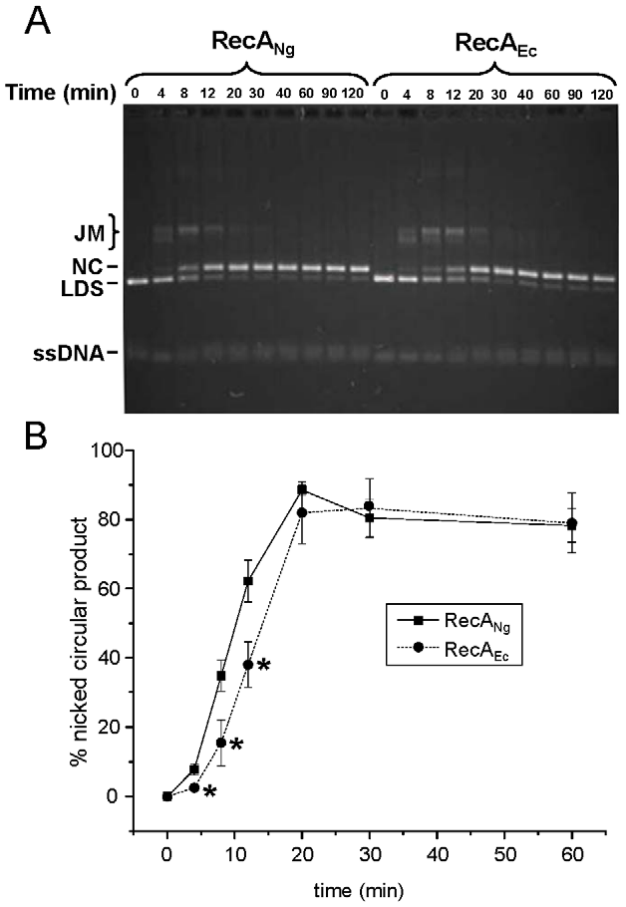
DNA strand exchange activity of RecA_Ng_ and RecA_Ec_ proteins. Reactions were carried out as described in the Materials and Methods and Results sections using cognate SSB proteins and the described substrates. Aliquots of the strand exchange reactions were removed and stopped at each indicated time point. The substrate linear dsDNA, joint molecule reaction intermediates, and nicked circular products are denoted LDS, JM, and NC, respectively. All ssDNAs (circular or linear), migrate identically under these gel conditions. A. RecA_Ng_ promotes faster strand exchange than RecA_Ec_ using homologous substrates. Representative gel of strand exchange reactions performed using homologous pGEM cssDNA and linear dsDNA and the cognate SSB proteins. B. Nicked circular product formation plotted versus time. Error bars represent the standard error of the mean of 4 separate experiments. *P<0.05 by Student's two-tailed *t*-test. (Note that not all time points shown in Figure 2A are represented on this graph.)

Recombination is essential for a number of cellular processes in *N. gonorrhoeae*; therefore, we assessed the recombinase activity of RecA_Ng_ by measuring the ability of RecA_Ng_ to catalyze the three-strand DNA exchange reaction relative to RecA_Ec_ *in vitro*. In this reaction, circular ssDNA and homologous linear dsDNA (LDS) molecules are incubated together in the presence of RecA, an ATP regeneration system, and SSB protein. RecA promotes pairing of the homologous molecules, yielding a joint molecule (JM), and subsequently transfers the complementary linear strand to the circular ssDNA by branch migration, yielding nicked circular (NC) and linear ssDNA products (Figure 2A). Using completely homologous *Nde*I-cut (generating a 5′ overhang) pGEM DNA substrates, RecA_Ng_ and RecA_Ec_ proteins, and the cognate SSB proteins in DNA strand exchange reactions, we observed that RecA_Ng_ formed significantly more NC product (*P*<0.05) than RecA_Ec_ at early time points (time 4, 8, 12 min) (Figure 2B). However, both RecA_Ng_ and RecA_Ec_ eventually yielded the same amount of nicked circular product (time 20, 30, 60 min) (Figure 2B). Similar results were seen for strand exchange reactions using *Pst*I-cut linear ds pGEM substrates (generating a 3′ overhang) and ΦX174 DNA substrates (data not shown). Together, these data clearly demonstrate that RecA_Ng_ promotes increased stand exchange at early time points, but does not show a higher overall efficiency of strand exchange relative to RecA_Ec_.

We characterized the binding activity of SSB_Ng_ to ssDNA and found it to be essentially the same as that of SSB_Ec_ (Figure S2). To measure the contribution of SSB to the efficacy of strand exchange, we carried out strand exchange reactions with both the cognate and the non-cognate SSB proteins and each of the RecA proteins. RecA_Ng_ showed the same kinetics of strand exchange with either SSB_Ng_ or SSB_Ec_, and RecA_Ec_ showed the same kinetics with either SSB_Ec_ or SSB_Ng_ (data not shown). Therefore, the increased efficiency of strand exchange appears to be strictly due to intrinsic features of the RecA_Ng_ protein.

### RecA_Ng_ promotes DNA strand exchange in the presence of lower Mg^2+^ than RecA_Ec_

**Figure 3:**
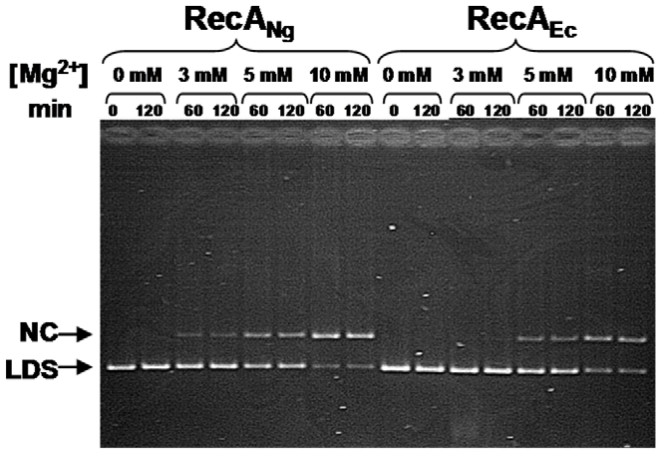
Strand exchange activity of RecA_Ng_ and RecA_Ec_ at varying levels of Mg^2+^. Reactions were carried out as described in Materials and Methods using completely homologous ΦX174 DNA with the indicated levels of Mg^2+^ present in the reactions. A representative gel shows aliquots of the strand exchange reactions that were removed and stopped at the times indicated. Nicked circular product (NC) and linear dsDNA (LDS) are noted.

It is well established that RecA_Ec_ shows optimal strand exchange at about 10 mM Mg^2+^, with no detectable strand exchange occurring at 3 mM Mg^2+^ [53], [54]. We measured the ability of RecA_Ng_ to catalyze strand exchange using homologous ΦX174 DNA substrates over a range of Mg^2+^ levels (Figure 3). Like RecA_Ec_, RecA_Ng_ showed the highest activity between 10–20 mM Mg^2+^ (Figure 3 and data not shown). However, unlike RecA_Ec_, RecA_Ng_ promoted a small amount of strand exchange at 3 mM Mg^2^. In the average of three independent experiments RecA_Ng_ catalyzed the conversion of significantly more ldsDNA substrate to the NC form (20% of DNA in NC form ± 1.5% standard error) than RecA_Ec_ (0% of DNA in NC form) after 60 min (*P* = 0.005 relative to RecA_Ec_ by Student's *t*-test) (Figure 3 and data not shown). These results demonstrate that, although this level of Mg^2+^ is not optimal for RecA_Ng_, activity, the protein can function, whereas RecA_Ec_ cannot.

### RecA_Ng_ shows higher ATPase activity than RecA_Ec_

We measured the DNA-dependent ATPase or dATPase activity of RecA_Ng_ relative to RecA_Ec_ in the presence of ssDNA using a coupled spectrophotometric assay [55], [56]. Briefly, cssM13mp18 DNA was incubated with either RecA_Ng_ or RecA_Ec_ for 10 min. Addition of either ATP (or dATP) and the cognate SSB protein started the reaction in which we measured the rate of (d)ATP hydrolysis by the decrease in absorbance at 380 nm (Table 1). RecA_Ng_ exhibited a k_cat_ of 44.74±1.10 min^−1^ which is approximately 1.5 times that of RecA_Ec_ in the presence of ATP and 10 mM Mg^2+^ and is the highest ATPase activity of any bacterial RecA protein described to date. The same trend is also observed when dATP is used as the nucleotide cofactor with RecA_Ng_ hydrolyzing dATP with a kcat of 59.66±0.58 min^−1^ when 10 mM Mg^2+^ are present. We tested whether RecA_Ng_ is operating at maximal velocity in our experiments by varying the concentrations of ATP in the reactions and found that the rates of ATP hydrolysis did not change with varying ATP concentrations (data not shown). Because of the noticeably high (d)ATPase activity of RecA_Ng_ we were interested in further characterizing the basis of this enhanced ATPase activity and possible ramifications for this enhanced ATPase activity on strand exchange.

**Table 1:** k*_cat_* of ATP or dATP hydrolysis by RecA_Ng_ and RecA_Ec_ in the presence of Mg^2+^.

*k*_cat_ of ATP hydrolysis [min^−1^]				
	RecA_Ng_		RecA_Ec_	
	10 mM Mg^2+^	3 mM Mg^2+^	10 mM Mg^2+^	3 mM Mg^2+^
ATP	44.74 (±1.10)	51.21 (±3.77)	28.25 (±0.88)	31.67 (±1.59)
dATP	59.66 (±0.58)	63.46 (±1.23)	40.38 (±2.24)	45.23 (±1.47)

In all experiments the concentrations of RecA protein and M13mp18 css DNA are 10 µM and 5 µMnt, respectively. k*_cat_* was calculated over a linear range of rates from 4 to 14 minutes for all experiments. Time zero represents the addition of 3 mM ATP and 0.5 µM SSB. Averages and standard deviations were calculated from values from 3 independent experiments.

### RecA_Ng_ is more efficient at displacing SSB from ssDNA than RecA_Ec_

**Figure 4:**
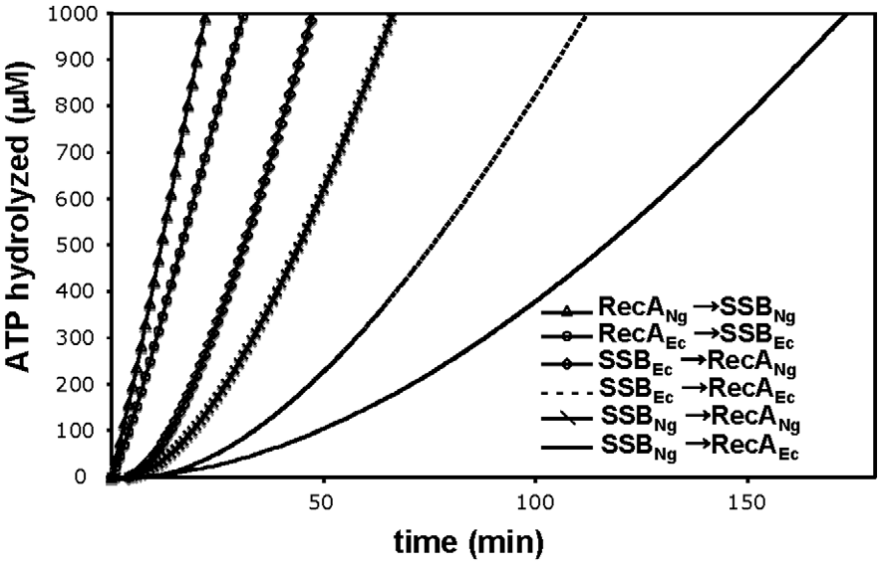
SSB displacement from ssDNA by RecA_Ng_ and RecA_Ec_. The experiments were carried out as described in Materials and Methods. The order of addition and the source of the proteins are indicated in the figure. Control reactions (Δ and O) contained 3 µMnt M13mp18 cssDNA, and 4 µM RecA. After 10 minutes of incubation, at t = 0, 3 mM ATP and 0.5 µM SSB were added to initiate the reaction. In all other reactions, 0.5 µM SSB was incubated with 3 µMnt M13mp18 cssDNA and 3 mM ATP for 10 minutes until 4 µM RecA was added at t = 0.

An additional protein component that could affect both the ATPase and recombinase activity of RecA_Ng_ is the SSB_Ng_ protein. The SSB_Ec_ protein has been shown to stimulate RecA_Ec_-promoted DNA strand exchange and ATPase activity [4], [57], as well as stimulate the activities of non-cognate recombinases [22], [58]. Moreover, orthologous SSB proteins from both bacteria [20], [21]and yeast [19] have been shown stimulate the reactions promoted by RecA_Ec_, suggesting that the action of SSB is not due to species-specific protein-protein interactions but likely due to melting of any secondary structure in the DNA by SSB. To determine whether the SSB proteins could differentially influence the ability of the RecA_Ng_ and RecA_Ec_ proteins to nucleate on ssDNA, we tested whether SSB presents a barrier to RecA_Ng_ nucleation and whether RecA_Ng_ can displace SSB more readily than RecA_Ec_. We carried out ATPase assays where SSB, ATP and cssDNA were incubated for ten minutes, allowing SSB to coat the DNA. These reactions were started by the addition of either RecA_Ng_ or RecA_Ec_ and required RecA to displace SSB to nucleate onto the DNA. RecA_Ng_ was more efficient than RecA_Ec_ at displacing both SSB_Ng_ and SSB_Ec_ (Figure 4). The lag-times for reaching steady state ATP hydrolysis for RecA_Ng_ on SSB_Ec_ or SSB_Ng_-coated DNA were 15.1±0.4 minutes and 23.7±0.7 minutes, respectively. RecA_Ec_ showed lag-times of 44.3±1.2 minutes and 73.0±5.6 minutes for displacing SSB_Ec_ or SSB_Ng_, respectively. These data not only show that RecA_Ng_ is faster at nucleating onto SSB-coated DNA than RecA_Ec_, but also that SSB_Ng_ is a greater barrier to RecA nucleation than is SSB_Ec_, perhaps due to unique features of the SSB_Ng_ protein that are manifest only in the ATPase assay, since the efficiency of strand exchange was not affected by replacing SSB (data not shown).

### The ATPase activity during homologous pairing is faster for RecA_Ng_ than for RecA_Ec_

**Figure 5:**
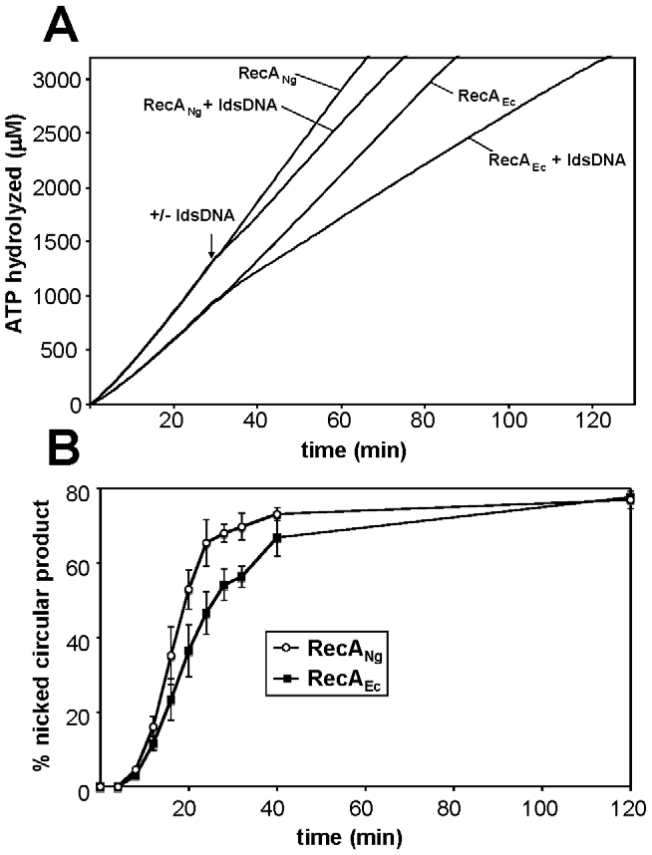
ATP hydrolysis by RecA_Ng_ and RecA_Ec_ during strand exchange. Reactions (510 µl) were carried out as described in Experimental Procedures and contained 4 µMnt M13mp18 cssDNA, 2.67 µM RecA_Ng_ or RecA_Ec_, 3 mM ATP, 0.4 µM SSB_Ng_ or SSB_Ng_ and 8 µMnt M13mp18 ldsDNA cut with *Pst*I. A) ATP hydrolysis during DNA strand exchange. Time t = 0 indicates the addition of ATP and SSB. Either ldsDNA or compensating TE storage buffer were added at t = 30 as indicated by the arrow. One representative graph of three reproducible experiments is shown. B) Nicked circular product formation plotted versus time. Time point 0 minutes represents the addition of ldsDNA to initiate strand exchange. The error bars are one standard deviation from the mean calculated from three independent experiments.

Although RecA_Ng_ exhibits a higher ATPase activity than RecA_Ec_ and catalyzes more strand exchange at early time points than RecA_Ec_, the difference in product formation during strand exchange is not as great as the elevated ATPase activity of RecA_Ng_ would suggest. Therefore, we examined whether the ATPase activity of RecA_Ng_ decreases to a similar degree as RecA_Ec_ ATPase activity during strand exchange. In RecA_Ec_, homologous pairing leads to a conformational change to the P-state, which characteristically shows decreased ATPase activity [5], [59], [60]. We carried out strand exchange reactions while monitoring RecA ATPase activity in a spectrophotometer (Figure 5A). Upon addition of linear dsDNA, the ATPase activity of RecA_Ng_ decreased by 20.3±4.3% whereas the ATPase activity of RecA_Ec_ decreased by 34.1±2.9% as shown in Figure 5A. The rate of ATP hydrolysis by RecA_Ng_ during strand exchange was similar to the ATPase activity of RecA_Ec_ on circular ssDNA, and greater than the ATPase activity of RecA_Ec_ during strand exchange. These data are all consistent with RecA_Ng_ forming more nicked circular product at earlier time-points than RecA_Ec_. Based on the greater ATPase activity of RecA_Ng_ compared to RecA_Ec_, we would have expected a more substantial difference between the two RecA proteins in product formation during strand exchange (Figure 5B). RecA_Ng_ forms at most 25% more nicked circular product than RecA_Ec_ at any given time-point, whereas the ATPase activity of RecA_Ng_ is roughly 40% higher than the ATPase activity of RecA_Ec_ during strand exchange. This comparison suggests that ATP hydrolysis and strand exchange are coupled differently in RecA_Ng_ and RecA_Ec_.

### DNA strand exchange activity using substrates to simulate the cellular processes of DNA transformation and antigenic variation *in vitro*

Our data thus far have demonstrated that RecA_Ng_ catalyzes more strand exchange at early time points and exhibits a more robust ATPase activity than RecA_Ec_ on DNA substrates commonly used in the laboratory, both in the context of stand exchange and alone. Since RecA recombinase activity is important for the lifestyle of *N. gonorrhoeae*, we wanted to test the hypothesis that RecA_Ng_ is more efficient than RecA_Ec_ at catalyzing strand exchange through substrates generated to mimic the cellular processes of DNA transformation and antigenic variation.

#### DNA transformation

**Figure 6:**
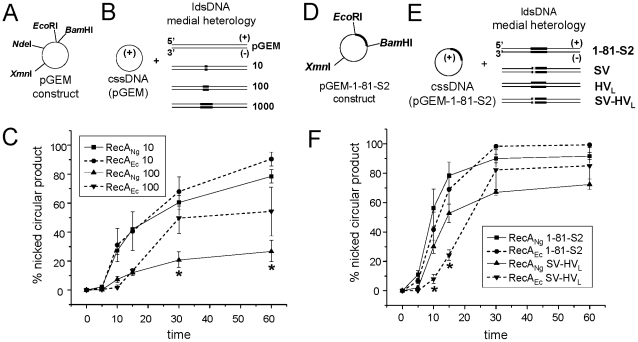
Strand exchange with DNA substrates to mimic DNA transformation and antigenic variation *in vitro*. A. Schematic of pGEM vector with relevant restriction sites used to clone heterologous inserts (see Materials and Methods). B. Linear dsDNA heterologous inserts digested with *Nde*I to give medial heterology and reacted with pGEM cssDNA. C. % nicked circular product observed in strand exchange reactions promoted by RecA_Ng_ and RecA_Ec_ using pGEM circular ssDNA reacted with pGEM-10 and pGEM-100 linear dsDNA (designated “10” and “100” in Figure). Error bars represent the standard error of the mean of 3 independent experiments. *P<0.05 by Students two-tailed *t*-test (RecA_Ng_ 100 compared to RecA_Ec_ 100). D. Schematic of construct pGEM 1-81-S2 containing the 1-81-S2 *pilE* DNA sequence and relevant restriction sites. E. Linear dsDNA of antigenic variants SV, HV_L_, and SV-HV_L_ heterologies (designated with shading and cross-hatches, see Figure S3 and Materials and Methods) digested with *Xmn*I to give medial heterology and reacted with pGEM 1-81-S2 circular ssDNA. F. % nicked circular product observed in strand exchange reactions promoted by RecA_Ec_ and RecA_Ng_ using pGEM-1-81-S2 cssDNA reacted with pGEM-1-81-S2 or pGEM-SV-HV_L_ linear dsDNA. Error bars represent the standard error of the mean of at least 3 independent experiments. *P<0.05 by Students two-tailed *t*-test (RecA_Ng_ SV-HV_L_ relative to RecA_Ec_ SV-HV_L_).

The ability of *N. gonorrhoeae* to take up and incorporate environmental DNA into its genome efficiently is important for the spread of antibiotic resistance genes [39]. Therefore we asked whether RecA_Ng_ is specifically adapted to allow the recombination of heterologous DNA into the gonococcal genome. We created a number of DNA substrates to mimic the DNA transformation of antibiotic resistance genes *i.e.* DNAs with increasing amounts of insert heterology: pGEM with either a 10 bp *Pac*I linker (pGEM-10), a 100 bp fragment of an *erm* ^R^ gene (pGEM-100), or a 1000 bp fragment of the *erm* ^R^ gene cloned into the vector (pGEM-1000) (Figure 6A, B). To evaluate the ability of RecA_Ng_ and RecA_Ec_ to promote strand exchange through DNAs with increasing heterology, pGEM circular ssDNA was isolated and reacted with the each of the heterologous dsDNA substrates. The dsDNA substrates were digested with *Nde*I, which places the region of DNA heterology in the middle of the linear dsDNA molecule (medial heterology) (Figure 6A,B). We observed that RecA_Ng_ and RecA_Ec_ exhibited essentially identical strand exchange ability through the heterologous pGEM-10 construct. Through the construct with 100 bp of heterology, pGEM-100, RecA_Ng_ exhibited slightly increased formation of NC product only at 10 minutes; however, RecA_Ec_ exhibited increased formation of NC product in later time points (time 30, 60) (Figure 6C). Neither RecA protein was able to yield the NC form when circular ss pGEM DNA was reacted with the linear ds pGEM-1000 DNA (data not shown). These data demonstrate that, despite the increased degree of strand exchange using completely homologous DNA substrates and the increased ATPase activity of RecA_Ng_, the RecA_Ec_ protein exhibits more strand exchange through substrates with a 100 bp heterologous DNA insert.

#### Pilin antigenic variation

Pilin antigenic variation occurs when regions of DNA located in silent, unexpressed *pilS* copies recombine unidirectionally into the *pilE* expression locus, resulting in a new pilin variant to be expressed on the surface of the gonococcal cell. We wanted to determine whether RecA_Ng_ is specifically adapted to catalyze recombination at *pilE*. To do this, we created DNA substrates to mimic the cellular process of antigenic variation *in vitro*. The 1-81-S2 variant *pilE* sequence, which was originally isolated during a human volunteer study [61], as well as three *pilE* variants of the 1-81-S2 sequence which arose during normal cultivation of the *N. gonorrhoeae* strain variant 1-81-S2 on agar plates [42], containing 7–22 variable residues (see Materials and Methods) were cloned into the pGEM vector (Figure 6D, E; Figure S3). To evaluate the ability of the RecA_Ng_ and RecA_Ec_ proteins to promote strand exchange through these *pilE* variant constructs, pGEM-1-81-S2 circular ssDNA was isolated and reacted with each of the four linear dsDNA constructs described above. dsDNAs were digested with *Xmn*I, which places the region of DNA heterology in the middle of the linear dsDNA molecule (medial heterology) (Figure 6D,E). RecA_Ng_ catalyzed more conversion to the NC form utilizing all four of the linear dsDNA substrates (time 10, 15 min) (Figure 6F and data not shown). Although RecA_Ec_ appeared to catalyze more overall conversion to NC product than RecA_Ng_, (time 30, 60), the differences were not statistically significant (Figure 6F and data not shown). These strand exchange data show that, at early time points, RecA_Ng_ catalyzes more strand exchange through regions of microheterology (7–22 variable residues in a 3000 bp piece of DNA), but that RecA_Ng_ does not yield more NC product over time. Taken together, the strand exchange data generated using more biologically relevant DNA substrates suggest that RecA_Ng_ is more efficient than RecA_Ec_ at catalyzing strand exchange through regions of microheterology, but that RecA_Ec_ is more efficient at catalyzing strand exchange through large heterologous inserts.

### RecA_Ng_-promoted cleavage of the LexA repressor

**Figure 7:**
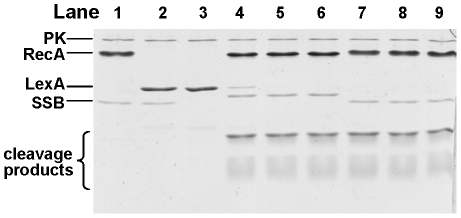
LexA cleavage promoted by RecA_Ng_ and RecA_Ec_. LexA was incubated with RecA proteins in the presence of cssDNA, an ATP regeneration system [pyruvate kinase (PK) and phosphor(enol)pyruvic acid], and the cognate SSB protein over a 30 minute time course. Reactions were stopped and visualized on a 17% SDS-PAGE stained with Coomassie Brilliant Blue. Lanes 1–3 are negative controls (incubated for 30 min) which lack various protein components of the reaction and are as follows: 1) lacks only LexA; 2) lacks RecA; 3) lacks RecA and SSB; lanes 4–6 contain the complete reaction and RecA_Ng_ and SSB_Ng_ proteins, with time points taken at 5, 15, and 30 min; lanes 7–9 contain the complete reaction and RecA_Ec_ and SSB_Ec_ proteins with time points taken at 5, 15, and 30 min. The two LexA cleavage products are visible at the bottom of the gel. The faint band in lane 3 that migrates slightly slower than the LexA cleavage products is likely a breakdown product of LexA.

*N. gonorrhoeae* lacks a classical SOS response. There is no upregulation of *recA* transcript or RecA protein following DNA damage [47], [48], and no homologs of the LexA, UmuC, or UmuD proteins predicted in the FA1090 genome [47]. However, recent work has revealed that *N. gonorrhoeae* encodes a LexA homologue that controls the expression of a small gene regulon [50]. To directly test whether RecA_Ng_ can act as a coprotease, we measured the ability of RecA_Ng_ to promote cleavage of the *E. coli* LexA protein. Both RecA_Ng_ and RecA_Ec_ promoted the cleavage of the 22 kDa LexA protein into two fragments of approximately 9 kDa and 13 kDa after 15 min. No cleavage of LexA was observed in the absence of RecA_Ng_ protein, demonstrating that cleavage is dependent on RecA_Ng_ (Figure 7). These results clearly show that RecA_Ng_ possesses coprotease activity sufficient to promote cleavage of the *E. coli* LexA protein *in vitro*.

## Discussion

Although several bacterial RecA homologs have been purified and characterized [22], [23], [58], [62]–[64], the vast majority of our understanding of RecA structure and function comes from studies of the *E. coli* RecA protein. Our goal for this work was to begin a biochemical characterization of the RecA protein from *N. gonorrhoeae*, which is important for a number of cellular processes related to pathogenesis. We show that the ATPase activity of RecA_Ng_ is the highest of any characterized bacterial RecA protein to date, with a k_cat_ min^−1^ of 44.7. Previous reports identified k_cat_ min^−1^ values ranging on the lower end of the spectrum between 10.0–13.0 for *B. subtilis* [22], *D. radiodurans* [58], *M. smegmatis*, and *M. tuberculosis* [65]; mid-range kcat min^−1^ values between 24.0–33.4 for *E. coli*, *Pseudomonas aeruginosa* [66], and *Salmonella typhimurium* [62], and the relatively high k_cat_ min^−1^ value of 40.0 for *S. pneumoniae* [22]. It is interesting to note that the two RecAs with the highest ATPase activities are *S. pneumoniae* and *N. gonorrhoeae*, both of which are naturally competent for DNA transformation. However, since the RecA from the naturally competent *B. subtilis* displays a relatively low k_cat_ min^−1^ of 10.0, enhanced ATPase activity is not a trait common to all naturally competent organisms.

Although certain steps of RecA-promoted DNA strand exchange are not absolutely dependent upon the ability of RecA to hydrolyze ATP, hydrolysis of ATP is required to yield strand exchange products greater than 1–2 kb, is necessary for bypass of heterologous DNA inserts, and is somehow coupled to the final extension step of DNA strand exchange [11]–[13]. It is likely that the coupling mechanism involves the end-dependent dissociation of RecA subunits from the 5′-proximal end of the RecA filament [60]. In addition to the increased ATPAse activity of RecA_Ng_ relative to RecA_Ec_, the conversion to the NC form in early time points by RecA_Ng_ was also significantly higher than that of RecA_Ec_ (Figure 2). We also observed small increases in strand exchange through substrates constructed to mimic the cellular process of antigenic variation *in vitro* (containing small microheterologies); however, RecA_Ng_ showed a lower ability to catalyze strand exchange through a 100 bp heterologous insert than RecA_Ec_ (Figure 6C). Interestingly, when the 100 bp heterology was located at the distal end of the lds DNA molecule, RecA_Ng_ catalyzed a significantly higher percent conversion to NC product than RecA_Ec_ (data not shown). Together, these results may suggest that ATPase activity and strand exchange are coupled differently in the RecA_Ng_ protein. It would be interesting to determine the whether the RecA proteins from other naturally competent bacteria also behave like RecA_Ng_ *in vitro*.

RecA_Ng_ converted a higher more ldsDNA to the NC form in early time points than did RecA_Ec_ when using completely homologous DNA substrates, but this did not result in a higher percentage of NC product in later time points. We previously conducted a cross-complementation study where the *recA* genes from *E. coli* and *N. gonorrhoeae* were expressed in *N. gonorrhoeae* from the same *lac* promoter construct (strains *recA*_Ec_ and *recA*_Ng_, respectively). That study revealed that strain *recA*_Ng_ showed 10-fold greater transformation efficiency than strain *recA*_Ec_ gene [67], when the transforming DNA contained a point mutation that renders recipient cells resistant to the antibiotic streptomycin. *recA*_Ng_ also showed a slightly, but not statistically significant, higher level of pilus phase variation relative to strain *recA*_Ec_. Taken together, these data suggest that RecA_Ng_ is more efficient at performing homologous recombination in the bacterial cell as well as *in vitro* through nearly homologous substrates. Additionally, since strain *recA*_Ec_ contained the endogenous gonococcal *ssb* gene, this may have also negatively influenced its ability to act as a recipient for DNA transformation and to perform antigenic variation. Our current data demonstrates that SSB_Ng_ is not displaced as well as SSB_Ec_ by RecA_Ec_ *in vitro* (Figure 4), although SSB_Ng_ does not inhibit the strand exchange ability of RecA_Ec_ relative to SSB_Ec_ (data not shown). Alternately, other unknown cellular factors may have influenced the ability of RecA_Ec_ to recombine DNA into the gonococcal genome or promote antigenic variation.

RecA_Ng_ also differed from RecA_Ec_ in its ability to function under low concentrations of Mg^2+^. We observed strand exchange by RecA_Ng_, but not RecA_Ec_ at 3 mM Mg^2+^ (Figure 3). Lusetti *et al*. showed that removal of the negatively charged C-terminus of RecA_Ec_ results in efficient strand exchange occurring at lower levels of Mg^2+^ (2–3 mM), presumably due to the loss of a C-terminal flap of the RecA protein that occludes binding of dsDNA in the absence of excess Mg^2+^ [68]. The C-termini of RecA proteins are characteristically divergent [52], and this is also the case with RecA_Ng_ and RecA_Ec_ (Figure 1). While both proteins contain 7 negatively charged amino acids in their 17 C-terminal residues, RecA_Ng_ also contains 2 positively charged amino acids (H323, H336), which could influence its requirement for magnesium. Alternately, the ability of RecA_Ng_ to function under lower levels of Mg^2+^ could also reflect a lower concentration of Mg^2+^ in the gonococcal cell, but this has not been experimentally determined.

In the current work, we showed that RecA_Ng_ promotes autocatalytic cleavage of the *E. coli* LexA protein *in vitro*. Although *N. gonorrhoeae* has historically been classified as lacking an SOS response [46], [47], *N. gonorrhoeae* in fact encodes a LexA-like protein, NG1427, which regulates expression of a small regulon in a *recA*-dependent manner in response to non-oxidative DNA damaging agents, but in a *recA*-independent manner in response to treatment with oxidative DNA damaging agents [50]. RecA_Ng_ and RecA_Ec_ proteins are both able to promote cleavage of the NG1427 protein *in vitro* [50]. Although a specific region of the *E. coli* RecA protein required for binding LexA repressor has not been identified, several individual residues have been implicated (Pro67, Glu154, Gly229, Arg243) [69]. These residues are all conserved in RecA_Ng_ (data not shown), which is consistent with the ability of RecA_Ng_ to cleave both LexA and NG1427. Interestingly, we have previously shown that heterologous expression of RecA_Ng_ in *E. coli* only partially complements an *E. coli recA* mutant for UV survival, and that this is only partly due to the inability of RecA_Ng_ to induce the *E. coli* SOS response [67]. Since RecA_Ng_ retains the ability to cleave LexA *in vitro*, there are a number of possibilities that could account for the differences observed *in vitro* versus in the cell. RecA_Ng_ may not interact efficiently with the LexA repressor in *E. coli* due to the presence of additional protein factors. This would result in only partial cleavage of the pool of LexA in *E. coli*, and subsequently weak induction of the SOS response. Alternately, RecA_Ng_ may not efficiently cleave the UmuD protein in the cell, which would also affect the survival of the *E. coli* strain carrying RecA_Ng_. Importantly, the RecA protein from the Gram-negative bacterium *S. pneumoniae* also facilitates autocatalytic cleavage of the *E. coli* LexA repressor *in vitro*, despite the fact that there is no LexA protein in the *S. pneumoniae* genome [22]. Taken together, these results suggest that the coprotease activity of RecA proteins may be dependent upon either the overall conserved structure of the RecA protein or residues which are important for other RecA activities, since coprotease activity is maintained in two distantly related bacteria which do not possess classical SOS systems.

In summary, the most remarkable aspects of the RecA_Ng_ protein are its robust ATPase activity and its increased ability to promote strand exchange at early time points. Although this increased ATPase activity did not render RecA_Ng_ better able to catalyze strand exchange through large regions of heterology, RecA_Ng_ did show an increased yield of NC product relative to RecA_Ec_ through regions of microheterology. These current data are consistent with a previous report showing that a *N. gonorrhoeae* strain carrying the *recA*_Ng_ gene exhibits higher levels of DNA transformation and antigenic variation, both of which are processes that rely on recombination through regions of microheterology, than an analogous strain carrying the *recA*_Ec_ gene [67]. Prior work also shows that the *recX* gene of *N. gonorrhoeae* is required for efficient antigenic variation and DNA transformation [30], but biochemical studies demonstrate that the RecX_Ng_ protein acts to limit the length of RecA_Ng_ nucleoprotein filaments to inhibit ATPase activity, suggesting that for optimal biological activity, the RecA_Ng_ nucleoprotein filament needs to be constrained by RecX_Ng_ [70]. Based on these findings, we postulate that the high ATPase activity of RecA_Ng_ has evolved to function with RecX_Ng_ control to facilitate antigenic variation and DNA transformation.

## Materials and Methods

### Enzymes, chemicals, and DNAs

The *E. coli* RecA, SSB [71], and LexA [72] proteins were purified as described. Restriction enzymes, Klenow, polynucleotide kinase (PNK), T4 DNA ligase, and M13K07 Helper Phage were purchased from New England Biolabs. *Pfu* and *Taq* enzymes were purchased from Invitrogen and Promega, respectively. Plasmid pGEM-3Zf(+) was purchased from Promega. Plasmid DNA purification and gel extraction kits were purchased from Qiagen. Pyruvate kinase, lysozyme, phosphoenolpyruvate, NADH, and ATP were purchased from Sigma. Isopropyl-1-thio-β-D-galactopyranoside was purchased from BioVectra or from Gold Biotechnology. Dithiothreitol was purchased from Research Organics, Inc. Unless otherwise noted, all other chemicals were purchased from Fisher.

### Buffers and media

Buffer P contained 20 mM potassium phosphate (pH 6.8), 1 mM DTT, 0.1 mM EDTA, and 10% (w/v) glycerol. Buffer R contained 20 mM Tris-Cl (80% cation) 1 mM DTT, 0.1 mM EDTA, and 10% (w/v) glycerol. *E. coli* strains were grown in liquid Luria-Burtani medium (LB) which contained 10 g/L trypone, 5 g/L yeast extract, and 10 g/L NaCl, with pH adjusted to 7.0, and LB agar additionally contained 15 g/L agar. *N. gonorrhoeae* strains were grown on Gc medium base (GCB; Difco) plus Kellogg supplements [22.2 mM glucose, 0.68 mM glutamine, 0.45 mM cocarboxylase, 1.23 mM Fe(NO_3_)_3_] [73] all from Sigma, at 37°C in 5% CO_2_.

### ATPase assay

A coupled spectrophotometric enzyme assay [56] was used to measure the DNA-dependent ATPase activities of RecA_Ng_ or RecA_Ec_. The regeneration of ATP from PEP and ADP was coupled to the oxidation of NADH and was observed as a decrease in absorbance at 380 nm (the maximal absorbance for NADH is at 340 nm, but 380 nm wavelength was used so that the signal remained within the linear range of the spectrophotometer for the duration of the experiment). The assays were carried out in a Varian Cary 300 dual beam spectrophotometer equipped with a temperature controller and a 12-position cell changer. The cell path length and band pass were 1 cm and 2 nm, respectively. The NADH extinction coefficient at 380 nm of 1.21 mM^−1^ cm^−1^ was used to convert the amount of NADH oxidized to the amount of ATP hydrolyzed. The reactions were carried out in a buffer containing 25 mM Tris-OAc (80% cation, pH 7.4), 1 mM DTT, 5% (w/v) glycerol, and 3 mM potassium glutamate. A concentration of 10 mM or 3 mM Mg(OAc)_2_ was added as indicated in Table 1. In reactions containing ATP, an ATP regeneration system consisting of 10 units/ml pyruvate kinase and 3.5 mM phosphoenolpyruvate was included in addition to a coupling system of 2 mM NADH and 10 units/ml lactate dehydrogenase. The concentrations of the regeneration system and coupling system enzymes in the presence of dATP were increased to final concentrations of 60 units/ml pyruvate kinase and 25 units/ml lactate dehydrogenase in order to eliminate lag-times of dATP hydrolysis. Reaction mixtures (100 µl) contained 5 µM circular M13mp18 ssDNA, 10 µM of RecA_Ng_ or RecA_Ec_ protein. The reactions were started after a 10-minute incubation of the reaction mixtures at 37°C by the addition of a mixture of ATP or dATP and SSB protein at 3 mM and 0.5 µM final concentrations, respectively.

### SSB-displacement ATPase assays

The ATPase assays were carried out as described under ATPase assays. Reactions were carried out in a buffer containing 25 mM Tris-OAc (80% cation, pH 7.5), 1 mM DTT, 5% (w/v) glycerol, 10 mM Mg(OAc)_2_ and 3 mM potassium glutamate. Reaction mixtures (100 µl) contained 3 µMnt M13mp18 cssDNA, 3 mM ATP and 0.5 µM SSB_Ec_ or SSB_Ng_. After 10 minutes of incubation at 37°C, RecA_Ng_ or RecA_Ec_ was added to a final concentration of 4 µM, representing t = 0. Control reactions were carried out where RecA was incubated with DNA and 10 minutes later ATP and SSB were added to start the reactions. As a quantitative measure for the time it takes RecA to reach steady state ATP hydrolysis, we use the x-intercept of an extrapolation of the linear portion of the line once steady state is reached to calculate a lag-time. A representative image is shown, but experiments were carried out in triplicate for calculation of lag-times.

### ATP hydrolysis during DNA strand exchange assays

The assays were carried out as described previously [60] with the following modifications. The circular double stranded M13mp18 DNA was completely digested with *Pst*I to generate the ldsDNA substrate. After initiating the reactions by addition of ATP and SSB, the reactions were incubated for 30 minutes at 37°C before ldsDNA was added, representing t = 30 in the ATP hydrolysis assay, but t = 0 in the strand exchange reaction. Stop points from the stand exchange reactions were taken at 0, 4, 8, 12, 16, 20, 24, 28, 32, 40 and 120 minutes by removing 15 µl of the reactions, and they were treated and analyzed as described [60]. Each reaction was carried out in triplicate.

### DNA substrates

Bacteriophage ΦX174 circular single-stranded DNA (virion) (ssDNA) was purchased from New England Biolabs. ΦX174 RFI supercoiled circular duplex DNA was purchased from Invitrogen. Linear duplex DNA (dsDNA) was generated by digestion of ΦX174 RFI with a variety of enzymes to yield blunt ends and 5′ overhangs. The digested DNA was extracted with phenol/chloroform/isoamyl alcohol (25∶24∶1) and ethanol precipitated. The cssDNA from bacteriophage M13mp18 (7249 nucleotides) was prepared as described [74], [75] with the following modifications. Chemically competent *E. coli* JM101 cells were transfected with commercially available, gel purified RFI M13mp18 DNA from NEB. The CsCl banding was done twice in a Beckman Ti60 rotor at 37,000 rpm for 15–20 hours. Concentrations of ssDNA and dsDNA were determined by absorbance with A_260 nm_ = 1 being equivalent to 36 and 50 µg mL^−1^, respectively. Molar concentrations of DNA are given in terms of total nucleotides.

### Creation of DNA transformation substrates

To create DNA substrates to mimic DNA transformation *in vitro*, heterologous inserts of increasing sizes were cloned into vector pGEM-3Zf(+). To create pGEM-Pac, pGEM-3Zf(+) was digested with *Sma*I, a 10-bp synthetic *Pac*I linker was ligated at the site, digested with *Pac*I, Qiaquick-purified to remove excess linker, and religated. To create pGEM-100 and pGEM-1000, 97- and 996-bp fragments of the *erm* ^R^ gene from pJD1145 [29] were generated by PCR using primer pairs ERM-1 (5′- CGCGGAATTCTCATGTTTGACAG-3′), which contains an *Eco*RI site (underlined) and ERM-3 (5′TTTCTCGTTCATTATAACCCTC-3′) or ERM-1 and ERM-2 (5′-GAAAGGTTGGGCTTCGGAATCG-3′). The resulting PCR products were treated with polynucleotide kinase (NEB), digested with *Eco*RI and gel purified using Qiaquick columns (Qiagen). The resulting fragments were directionally cloned into *Eco*RI-*Sma*I digested, CIP-treated pGEM-3Zf(+). All substrates were sequenced to verify insertion and orientation of the inserts.

### Creation of pilin antigenic variation substrates

To create DNA substrates to mimic pilin antigenic variation *in vitro*, we utilized previously-identified variants of *N. gonorrhoeae* with distinct changes at the *pilE* locus that had arisen during cultivation of *N. gonorrhoeae* [42]. *pilE* sequence variants with specific nucleotide changes in the semivariable (SV), hypervariable loop (HV_L_), and both regions (SV-HV_L_) (compared to the 1-81-S2 starting *pilE* sequence) were chosen. *pilE* sequences from individual colonies of *N. gonorrhoeae* propagated on GCB media were PCR-amplified using *Taq* polymerase and primers CONSTF2 and SP3A [61], blunt-ended with *Pfu* polymerase and treated with PNK, gel-purified using Qiaquick columns, and cloned into *Sma*I-digested, CIP-treated pGEM3Zf(+). Clones were sequenced to verify that only the desired changes were present in the *pilE* locus and that the *pilE* sequences were all present in the same orientation in vector pGEM3Zf(+).

### Purification of dsDNA and circular ssDNA from pGEM constructs

dsDNA and cssDNA was purified from *E. coli* strain JM109 carrying pGEM alone and the various pGEM constructs described above. dsDNA was purified using the Midiprep protocol recommended in the Qiagen manual. cssDNA of pGEM constructs was isolated according to the protocol recommended by NEB with modifications as follows: 250 mL of LB/Amp (75 µg/mL) was inoculated with a loopful of fresh colonies and grown at 37°C with vigorous aeration to A_600_<0.05. M13KO7 Helper Phage (final concentration 1×10^8^ pfu/mL) was added and cultures were grown an additional 90 min. Kanamycin (70 µg/mL) was added and cultures were grown overnight (18 h) with vigorous aeration at 37°C. Culture supernatants were purified from cells by two successive rounds of centrifugation at 12,000 x *g* for 15 min. After the first spin, the supernatant was transferred to a new tube. After the second spin, the supernatant was treated with DNaseI (Worthington) at 37°C for 15 min to digest any contaminating dsDNA, after which the upper 90% of the supernatant was transferred to a new tube. 0.2 volumes of 2.5 M NaCl/20% PEG was added and incubated at 4°C for 60 min, and cssDNA was recovered by centrifuging at 12,000 x *g* for 15 min. The supernatant was decanted and spun again briefly. The pellet was resuspended in 3.2 mL TE, transferred to microfuge tubes, and spun 13,000 x *g* for 5 minutes in a microcentrifuge to pellet any remaining cells. 400 µL of 2.5 M NaCl/20% PEG solution was added, incubated at room temperature for 5 min, and spun in a microfuge at 13,000 x *g* for 10 min. Supernatant was removed and tubes were spun again briefly to remove all traces of supernatant. Pellets were resuspended in 600 µL TE and extracted with successively with phenol (allowing for sit for 15 min before spinning), twice with phenol/chloroform/isoamyl alcohol (25∶24∶1), and once with chloroform/isoamyl alcohol (24∶1 v/v). ssDNA was precipitated with 2.5 M NaOAc and 100% EtOH, followed by a 70% EtOH wash. Dried pellets were resuspended in TE buffer and DNA quality was assessed by agarose gel. DNAs were quantified by spectrophotometer as described above.

### *N. gonorrhoeae recA* cloning

The *N. gonorrhoeae recA* gene was subcloned from construct pVD300*recA6* [76] into pET21a (Novagen). Plasmid pVD300*recA6* was used as a template in a PCR with an upstream primer consisting of a *Nde*I site and the first 24 bases of the *recA*_Ng_ gene. The ATG bases in the *Nde*I site are the first bases of the *recA*_Ng_ gene. The downstream primer consisted of a *Hind*III site followed by the last 21 bases of *recA*_Ng_. The coding for the proline 3 amino acids from the end was changed to CCG for better codon usage in *E. coli*. The PCR product was digested with *Nde*I and *Hind*III and inserted into the overproduction vector pET21a (Novagen) cut with the same restriction enzymes. The resulting plasmid was designated pEAW375. The presence of wild type *recA*_Ng_ was confirmed by direct sequencing.

### *N. gonorrhoeae* RecA (RecA_Ng_) purification

RecA_Ng_ protein was overexpressed and purified using modifications to the existing *E. coli* RecA purification protocol [71]. All steps were carried out at 4°C. Cell paste (22.6 g) was thawed and resuspended overnight in 75 mL of a solution consisting of 25% (w/v) sucrose and 250 mM Tris-Cl (80% cation, pH 7.5). Cells were lysed by 60 min incubation with 40 mL of a 5 mg/mL solution of lysozyme in 250 mM Tris-Cl (80% cation, pH 7.5), followed by addition of 0.4 mL of 25 mM EDTA per mL of solution, sonication, and centrifugation. The lysate was precipitated by dropwise addition of 22.2 mL of 5% (w/v) polyethyleneimine (pH 7.5) (0.05% final concentration), with constant stirring, and centrifuged. The resulting pellet was washed with 50 mL of R buffer plus 150 mM ammonium sulfate. RecA_Ng_ protein was extracted from the pellet by washing twice with 300 mM ammonium sulfate. Extract was precipitated with solid ammonium sulfate to 48% saturation and centrifuged. RecA_Ng_ protein was precipitated from the supernatant with solid ammonium sulfate to 80% saturation and centrifuged. It is to be noted that in a second preparation of RecA_Ng_, most of the RecA_Ng_ precipitated in the 48% ammonium sulfate precipitation. The pellet was washed two times with R+ 0.49 g/mL ammonium sulfate, resuspended in R plus 50 mM KCl and dialyzed against the same. RecA_Ng_ protein was loaded onto a DEAE-Sepharose column and washed with two column volumes of R buffer plus 50 mM KCl. Protein was eluted using a linear gradient of R + 50 mM to 1 M KCl over 10 column volumes. Peak fractions were identified by SDS-PAGE analysis, pooled, and dialyzed against 300 mM phosphate buffer. Protein was then loaded onto a hydroxyapatite column, washed with two column volumes of 300 mM phosphate buffer, and eluted with a linear gradient from 300 mM to 1 M phosphate over ∼3 column volumes. Peak fractions were identified by SDS-PAGE analysis, pooled, and dialyzed against R + 50 mM KCl. The following steps were employed in the instances when the protein was not nuclease free after the hydroxyapatite column: The RecA_Ng_ containing fractions from the hydroxyapatite column were concentrated by precipitation with 0.49 g/mL of solid ammonium sulfate, resuspended in 6 mL of R buffer plus 1 M KCl and loaded onto a Sephacryl S-300 16/60 gel filtration column. The protein was eluted by running one column volume of R plus 1 M KCl buffer through the column and the peak fractions were identified by SDS-PAGE. Pooled fractions were dialyzed against R plus 50 mM KCl and flowed through two subsequently hooked up 1 mL CM sepharose and SP sepharose cation exchange columns. The flow through was collected and directly loaded onto a Source 15 Q anion exchange column, washed with 2 column volumes of R plus 50 mM KCl and eluted with a 20 column volume linear gradient from R plus 50 mM KCl to R plus 1 M KCl. Peak fractions were identified by SDS-PAGE, pooled and dialyzed against R plus 50 mM KCl. If necessary, the protein was then concentrated with Centricon-Plus 20 10,000-Dalton molecular weight cut-off concentrators (Amicon), flash frozen in liquid N_2_, and stored at −80°C. The concentration of the RecA_Ng_ protein was determined from the absorbance at 280 nm using the calculated extinction coefficient 2.49×10^4^ M^−1^ cm^−1^. RecA_Ng_ protein was free from detectable nuclease activities.

### *N. gonorrhoeae ssb* cloning

The Gc *ssb* gene was amplified using primers GcSSBNdeI, which introduces a *Nde*I site (underlined) (5′-ACTGCATATGTCATTGAACAAAGTCATCC-3′) and GcSSB-2 (5′-GTAAAATTCAGAACGGGATGTCG-3′) using *Pfu* polymerase (Stratagene). The gel-purified PCR product was ligated to pCR-Blunt (Invitrogen) and clones were sequenced to verify that no mutations had been introduced. The *ssb* gene was excised from this construct by *Nde*I-*EcoR*I digestion and ligated to *Nde*I-*EcoR*I-digested pET21a (Novagen).

### *N. gonorrhoeae* SSB (SSB_Ng_) purification

SSB_Ng_ was overexpressed in BL21(DE3). All steps were carried out at 4°C. Cell paste (36 g) was thawed and resuspended overnight in 120 mL of a solution consisting of 0.2 M NaCl, 15 mM spermidine tri-Cl, 1 mM EDTA, 10% (w/v) sucrose and 50 mM Tris-HCl (pH 8.3). Cells were lysed by a 45 min incubation with lysozyme solution at a 0.2 mg/mL final concentration in the presence of 150 µl of 0.1 M PMSF. A 4% Na deoxycholate solution was made fresh (125 mM NaCl, 4% Na deoxycholate) and added to a final concentration of 0.05% followed by an incubation at room temperature for 10–15 min. The sample was sonicated for 5 one minute cycles with a 0.5 sec pulse at 60% output and then centrifuged at 16,000 rpm (38400 x *g*) for 110 minutes. To the supernatant, 10% polyethyleneimine was added (0.053 mL per mL of supernatant) followed by centrifugation at 10,000 rpm for 15 min. The resulting pellet was resuspended in 100 mL of TGE (50 mM Tris-HCl (39% cation, pH 8.3), 1 mM EDTA, 20% glycerol, 1 mM DTT)/0.4 M NaCl, stirred for 30 min and spun at 9000 rpm (12,156 x *g*) for 15 min. This step was repeated to get better extraction from the pellet. The two supernatants were combined and solid ammonium sulfate was added to 27% saturation (150 g/L). The solution was spun down at 16,000 rpm (38,400 x *g*) for 40 min. The pellet was washed with 50 mL of TGE/0.4 M NaCl/1.68 M ammonium sulfate and the solution was spun down at 16,000 rpm (38,400 x *g*) for 30 min. The resulting pellet was resuspended in 50 mL of TGE/0.4 M NaCl and dialyzed vs 1 L of TGE/0.2 M NaCl, followed by 2×1 L of TGE/0.05 M NaCl. The sample was loaded onto a HiTrap Q column (CV = 5 mL) and a 20 CV gradient was run from TGE/0.05 M NaCl to TGE/0.48 M NaCl. SSB_Ng_ eluted in the gradient, peak fractions were analyzed by SDS-PAGE and pooled. The protein was precipitated by the addition of solid ammonium sulfate to 40% saturation (242 g/L) and stirred over night. The precipitated solution was centrifuged for 18,000 rpm (39,191 x *g*) for 40 min. The pellet was resuspended with 1.75 mL of TGE/1 M NaCl and loaded on to a Sephacryl S100 HR 16/60 column that was equilibrated with the same buffer. The pooled fractions were dialyzed vs 3×1 L of TGE/0.1 M NaCl and loaded onto a ssDNA cellulose column equilibrated with the same buffer. The protein was eluted with TGE/1 M NaCl and the peak fractions were analyzed by SDS-PAGE, collected and dialyzed vs TGE/0.1 M NaCl. The protein was loaded onto a DEAE FF column (CV = 25 mL) and eluted in a 10 CV gradient from TGE/0.1 M NaCl to TGE/0.8 M NaCl. The peak fractions were analyzed by SDS-PAGE, and tested for nuclease contamination individually. The fractions that were free of detectable nucleases were pooled and dialyzed vs 3×2 L of SSB storage buffer (20 mM Tris-Cl (39% cation, pH 8.3), 0.5 M NaCl, 1 mM EDTA, 50% (v/v) glycerol, 1 mM DTT), and frozen with liquid N_2_ and stored at −80°C. The concentration was determined using the extinction coefficient for denatured protein of 22900 M^−1^cm^−1^.

### Electrophoretic mobility shift assay with SSB proteins

Fluorescent [5′-6FAM (6-carboxyfluorescein)], OCN324 50-mer DNA oligonucleotide (5′ to 3′ TGCCTCGCGGTAGCTCTTCTCGGAGCGCACGATTCGCACTGCTGATGTTC) was ordered from IDT (Integrated DNA Technologies Inc.). 50 µM (in nucleotides) of the oligonucleotide was incubated with various concentrations of SSB_Ec_ and SSB_Ng_ ranging from 10 nM to 10 µM in 20 mM Tris-Cl 39% cation (pH 8.3), 0.5 M NaCl, 1 mM EDTA, and 50% glycerol for 10 minutes at 37°C. The samples were then run on a 4% native PAGE for 2 hours at 150 V at 4°C. The positions of the fluorescent oligonucleotides were analyzed using a Typhoon scanner (Amersham Biosciences).

### DNA strand exchange reactions

The RecA-dependent DNA strand exchange reaction was carried out as described [57] between circular ssDNA (NEB) and linear dsDNA (Invitrogen) derived from ΦX174, M13mp18, or between the circular ssDNA and linear dsDNA substrates generated to mimic DNA transformation or pilin antigenic variation. Unless otherwise stated, all reactions were carried out at 37°C in solutions containing 25 mM Tris-acetate (80% cation, pH 7.5), 1 mM DTT, 5% glycerol, 3 mM potassium glutamate 10 mM magnesium acetate, and an ATP-regeneration system (10 units/mL of pyruvate kinase/3.3 mM phosphoenolpyruvate). For the reaction, the RecA_Ng_ or RecA_Ec_ protein was preincubated with 10 µM ΦX174 circular ssDNA for 10 min. SSB_Ec_ or SSB_Ng_ protein (1 µM) and ATP (3 mM) were then added, followed by an additional 10 min of incubation. ΦX174 linear dsDNA (10 µM) was added to start the reaction. Aliquots were removed from reactions and stopped by addition of 5 µL of a solution containing 15% Ficoll, 0.24% bromophenol blue, 0.24% xylene cyanol, and 4% SDS. Samples were subjected to electrophoresis using 0.8% agarose gels and 1 x TAE buffer, stained with ethidium bromide, and exposed to UV light using a Biorad Molecular Imager Gel Doc XR System gel imaging system. Gel images were analyzed using the compatible Quantity One software to determine %NC product of total DNA (LDS+NC+JM).

### RecA protein-promoted LexA cleavage assay

Reactions were carried out at 37°C in solutions containing 25 mM Tris-OAc (80% cation, pH 7.4) 1 mM DTT, 5% (w/v) glycerol, 3 mM postassium glutamate, 3 mM Mg(OAc)_2_, and an ATP regeneration system (2 mM phosphoenolpyruvate, 10 units/mL pyruvate kinase). The RecA_Ng_ or RecA_Ec_ proteins (3 µM) were preincubated with 9 µM ΦX174 circular ssDNA for 5 min. SSB_Ng_ or SSB_Ec_ proteins (0.9 µM) and ATP (3 mM) were added and reactions were incubated a further 5 min. LexA protein (3 µM) was added to start the reaction, which continued for 5–30 min. Time points were removed and added to Laemmli sample buffer (250 mM Tris-Cl pH 6.8, 4% SDS, 20% w/v glycerol, 10% 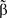-mercaptoethanol, and 0.1% w/v bromophenol blue) to stop the reaction. Samples were subjected to SDS-PAGE electrophoresis on 17% acrylamide gels and stained with Coomassie Brilliant Blue to visualize LexA cleavage.

## Supporting Information

Figure S1 **Purification of RecA_Ng_.** RecA_Ng_ was purified as described in Material and Methods and Results to >99.9% homogeneity. Aliquots of uninduced *E. coli* culture, induced *E. coli* culture, and the final purified RecA_Ng_ protein product were run on an SDS-PAGE gel and visualized by Coomassie blue stain.
(PDF)

Figure S2 **Electrophoretic mobility shift assay indicates SSB_Ec_ and SSB_Ng_ bind to ssDNA with similar affinity.** Increasing concentrations of SSB_Ec_ and SSB_Ng_ from 10 nM to 10 µM (corresponding to ratios of 1/5000 and 1/5 SSB monomers, or 1/20,000 and 1/20 tetramers to total nucleotides, respectively) were incubated with 50 µM nucleotides ssDNA fluorescent oligonucleotide and loaded onto a 4% native PAGE. The ssDNA is fully bound when SSB_Ec_ or SSB_Ng_ is present at a ratio of 1/10 SSB per base pair.
(PDF)

Figure S3 **DNA sequence alignment of** ***pilE*** **substrates cloned into pGEM.** Bases that differ from the parental 1-81-S2 sequence are shown in white.
(TIF)
