# Supplementary figures and images for: Purification and Characterization of the RecA Protein from Neisseria gonorrhoeae
Source: PLoS One. 2011 Feb 17;6(2):e17101. doi: 10.1371/journal.pone.0017101 (PMC3040777; doi:10.1371/journal.pone.0017101)

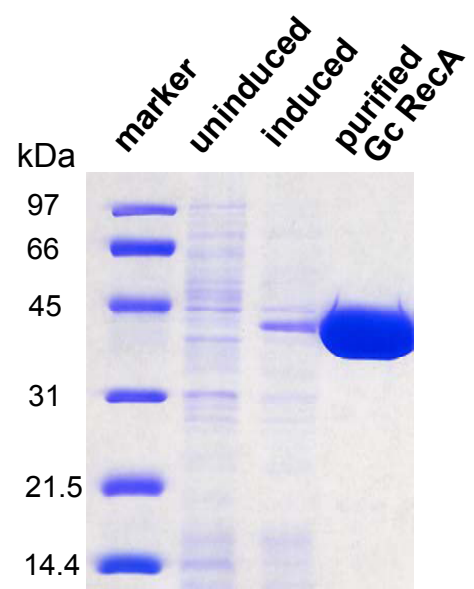

Supplement: Figure S1 — Purification of RecANg. RecANg was purified as described in Material and Methods and Results to >99.9% homogeneity. Aliquots of uninduced E. coli culture, induced E. coli culture, and the final purified RecANg protein product were run on an SDS-PAGE gel and visualized by Coomassie blue stain. (PDF) [file pone.0017101.s001.pdf]

***Escherichia coli* SSB and *Neisseria gonorrhoeae* SSB EMSA on ssDNA OCN 324 50mer**

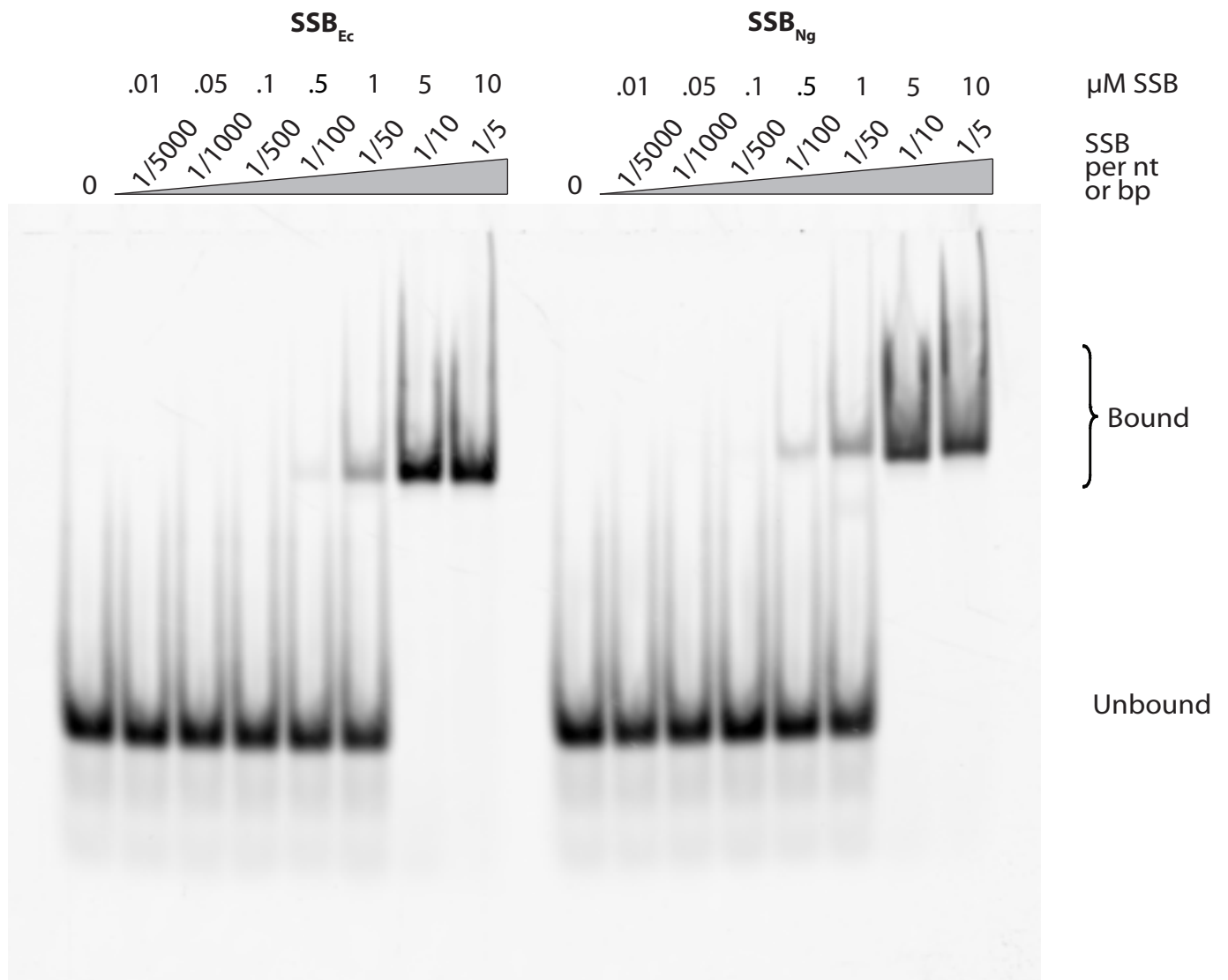

Supplement: Figure S2 — Electrophoretic mobility shift assay indicates SSBEc and SSBNg bind to ssDNA with similar affinity. Increasing concentrations of SSBEc and SSBNg from 10 nM to 10 µM (corresponding to ratios of 1/5000 and 1/5 SSB monomers, or 1/20,000 and 1/20 tetramers to total nucleotides, respectively) were incubated with 50 µM nucleotides ssDNA fluorescent oligonucleotide and loaded onto a 4% native PAGE. The ssDNA is fully bound when SSBEc or SSBNg is present at a ratio of 1/10 SSB per base pair. (PDF) [file pone.0017101.s002.pdf]

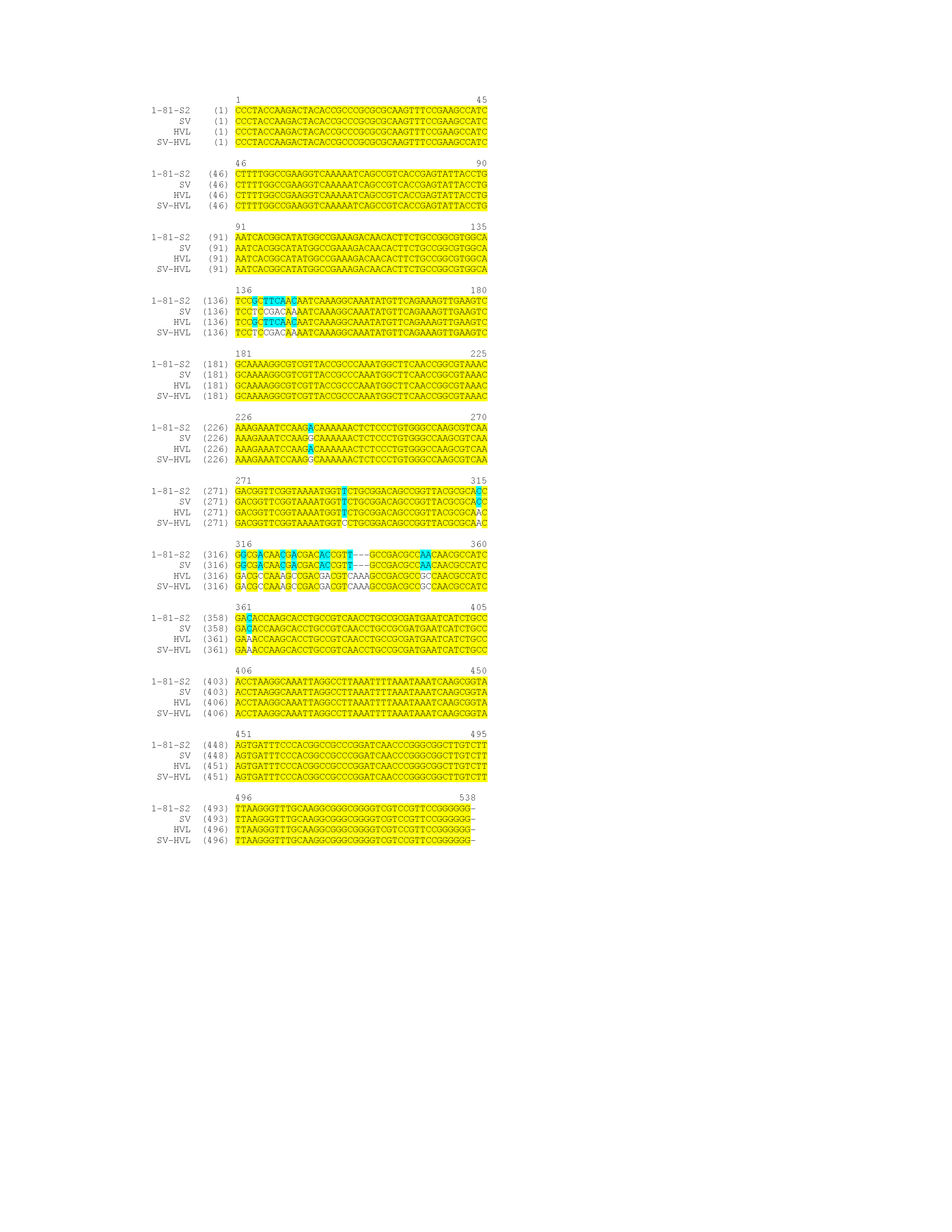

Supplement: Figure S3 — DNA sequence alignment of pilE substrates cloned into pGEM. Bases that differ from the parental 1-81-S2 sequence are shown in white. (TIF) [file pone.0017101.s003.tif]
